# Comparative Analysis of Classic Brain Component Sizes in Relation to Flightiness in Birds

**DOI:** 10.1371/journal.pone.0091960

**Published:** 2014-03-17

**Authors:** Matthew R. E. Symonds, Michael A. Weston, Randall W. Robinson, Patrick-Jean Guay

**Affiliations:** 1 Centre for Integrative Ecology, School of Life and Environmental Sciences, Deakin University, Burwood, Victoria, Australia; 2 Applied Ecology Research Group & Institute for Sustainability and Innovation, College of Engineering and Science, Victoria University, St. Albans, Victoria, Australia; University of Lethbridge, Canada

## Abstract

Increased encephalization has been linked to a range of behavioural traits and scenarios. However, studies of whole brain size in this context have been criticised for ignoring the role of specific brain areas in controlling behaviour. In birds, the response to potential threats is one such behaviour that may relate to the way in which the brain processes sensory information. We used a phylogenetic generalised least squares (PGLS) analyses, based on five different phylogenetic hypotheses, to analyse the relationship of relative sizes of whole brain and brain components with Flight-Initiation Distance (FID), the distance at which birds flee from an approaching human, for 41 bird species. Starting distance (the distance at which an approach to a bird commences), body mass and eye size have elsewhere been shown to be positively associated with FID, and consequently were included as covariates in our analysis. Starting distance and body mass were by far the strongest predictors of FID. Of all brain components, cerebellum size had the strongest predictor weight and was negatively associated with FID but the confidence intervals on the average estimate included zero and the overall predictor weight was low. Models featuring individual brain components were generally more strongly weighted than models featuring whole brain size. The PGLS analyses estimated there to be no phylogenetic signal in the regression models, and hence produced results equivalent to ordinary least squares regression analysis. However analyses that assumed strong phylogenetic signal produced substantially different results with each phylogeny, and overall suggest a negative relationship between forebrain size and FID. Our analyses suggest that the evolutionary assumptions of the comparative analysis, and consideration of starting distance make a profound difference to the interpretation of the effect of brain components on FID in birds.

## Introduction

Birds encounter an array of visual stimuli, some of which are benign (e.g. vegetation moving in the wind, or passing recreationists) and some of which are dangerous (e.g. approaching hunters or predators). Like all animals, birds make complex decisions regarding when and how to respond to potential threats [1]. Inappropriate responses may result in death or unnecessary disruption to normal activities and an associated deleterious change in energy budgets. Appropriate responses increase survival and fitness [2]. In behavioural research, a widely adopted measure of response is ‘Flight-Initiation Distance’ (FID), the distance at which birds flee from an approaching human [1]. Birds adjust their FIDs in relation to a range of factors, including body mass, encounter rates with stimuli, and aspects of the stimulus such as starting distance (the distance at which a human approach begins), stimulus type (e.g. vehicle or walker), proximity to refuge, directness and speed of approach [3].

The ability to discriminate between stimuli within species [4], [5] demonstrates that cognition is involved in the specifics of bird escape, and the substantial cognitive ability of at least some birds has recently been highlighted [6]. Accurate judgement of risk, and appropriate mediation of response, is likely to be critical for the survival of many birds that encounter potentially threatening stimuli such as humans in increasing numbers and places [7], [8]. The “cognitive buffer” hypothesis suggests larger-brained birds will be better able to adapt to novel environmental conditions, such as those created by anthropogenic landscape change [9]. In theory, these birds may be able to more accurately judge risk when presented with a stimulus, or be able to learn (habituate or sensitize) to adjust responses appropriately based on their previous experience [10]. If so, one would predict that there would be a negative association between FID and brain size within and across species, with larger brained birds being less ‘flighty’ as a response of learned habituation to non-threatening human stimuli.

Relative whole brain size is often used as a surrogate for a species' cognitive ability [9], [11]-[14], and is positively associated with, for example, improved survival and naturalisation success, and increased rates of behavioural innovation [9], [15], [16]. In the case of FID, an analysis of shorebirds found no link to whole brain size [10], although a previous study of urban bird species identified a positive association when considering intraspecific variability in FID [17]. Relative whole brain size is a convenient measure, because it can be estimated from endocranial volume, which is available from a large number of species of birds from various taxa [14], [15], [18]. However, it has been criticised as a measure of cognitive ability because of the likely functional specificity of brain components [11]. The ‘mosaic model’ of brain evolution suggests that selection should only act on brain components that are directly involved in mediating specific behavioural functions [19], and recent work on mice does indeed suggest that selection for particular behaviours can have direct consequences for the evolution of size of key brain components [20].

In birds the detection of a potential threat is likely to involve vision and perception (the optic lobe, forebrain and cerebellum), complex assessment of risk (the forebrain), and physiological and motor responses (brain stem, forebrain and cerebellum) [21]–[23]. Consequently we can make specific predictions in regard to the relationship of individual brain components to FID. For example, because eye size is positively associated with FID [24], and larger eye size requires large brain size to deal with processing of visual information [25], we might predict a positive association between optic lobe size and FID (although the association between optic lobe size and overall brain and eye size remains unclear). Conversely, in birds the cerebellum is associated with cognition [26], [27]. Since learning affects flight initiation responses [28], specifically to humans in the form of habituation [3], [29], then we might predict that species with larger cerebella, and hence greater capacity for learning, should show decreased FIDs in response to human approaches. Similarly, the capacity to respond more quickly may reduce FID, and hence a negative association between FID and brain stem size might be predicted. The link between forebrain size and FID is more difficult to predict, since it is involved in both perception and cognitive assessment, but existence of any association may provide insights into its role in flight initiation responses.

Brain components interact in complex ways and brain components exhibit multi-functionality, yet the need to analyse the influence of brain components on relevant aspects of life history remains [30]. Here we analyse the relationship between these brain components and flight-initiation distances for a sample of 41 bird species. The study follows a recent larger comparative analysis of 64 species by Møller and Erritzøe [31] which found that species with larger brains generally had smaller FIDs, but that relative cerebellum size was *positively* associated with FID (after controlling for eye size and body mass). Our analysis differs in several respects. First we employ a phylogenetic comparative method (phylogenetic generalised least squares) that explicitly assesses and controls for the estimated amount of phylogenetic signal in the data (Møller and Erritzøe used an independent contrasts approach which assumed that the phylogenetic effect was strong). Second we repeat the analyses using five different phylogenetic hypotheses to investigate variation in results dependent on the phylogeny used as the basis for analysis. Third we employ an information-theoretic model selection approach to identify the best models predicting FID and the relative importance of putative predictor variables. Finally our analyses also control in a different way for the confounding effect of starting distance (the distance at which an experimental approach to a bird begins) on FID. By comparing our results with those of this other recent study [31], we can provide a different insight into the factors which may determine the nature of the relationship of brain components to FID in birds.

## Materials and Methods

### Comparative data

We collated data on behaviour, morphology and brain regions - forebrain, cerebellum, optic lobe (comprising optic tectum and underlying structures such as inferior colliculus) and brain stem - for 41 bird species from the literature. In addition to relative size of brain components, a number of other prominent factors are known to positively influence FID, specifically starting distance (the distance from the focal bird at which an ‘approach’ begins) [32]–[34], body mass [3] and eye size [24], [31]. Thus, we include these variables in our analyses. We obtained FID and associated starting distances from Møller *et al.* [35]. These data were augmented by including data from rural birds [36] and from Blumstein [1]. Body masses were obtained from Dunning [37] supplemented by data on Australian sub-species from Marchant and Higgins [38]. Data for eye size (eye volume) were taken from Møller & Erritzøe [24], [31].

Information on the size of components of the avian brain is scarce. The size of the different regions of the brain was obtained from Portmann [39]. Absolute mass of the different regions of the brain was calculated by multiplying Portmann's “*Indices intra-cérébraux*” (Intra-cerebral Indices) by the “*chiffre basal*” (basal comparison number, the predicted mass of the brain stem of a Galliformes bird of the same mass) for each species as used in previous studies of brain components [31], [40]. Overall brain size was calculated as the sum of the mass of all the different regions. Raw data are presented in Table S1. We use the ‘classic’ nomenclature for brain components because it was available for a range of species, although we acknowledge that more recent advances in the understanding of the avian brain, would improve the interpretation of functionality of different areas [41], [42].

All variables were log10-transformed prior to analysis to better conform with assumptions of normality.

### Phylogenetic information

As with any cross-species comparative analysis, it is necessary to control for the effect of phylogenetic relationships [43], [44]. In phylogenetic comparative analyses, estimation of relationships may depend on the exact reconstruction of phylogeny used [45]. Consequently, we decided to repeat our comparative analyses using five different phylogenies (available in nexus file format in File S1).

The first two of these phylogenies were composite trees constructed from recently published molecular phylogenies of birds. For relationships within Passeriformes (the majority of species in our analysis) we used Hugall and Stuart-Fox [46]'s phylogeny (a pruned version supplied by Andrew Hugall, *pers. comm.*). Higher-level relationships between the other bird groups in the analysis were taken from the large inter-familial phylogeny of Hackett et al. [47], and further information on relationships within key groups was obtained from the following phylogenies: Charadriiformes [48], Galliformes [49], Gruiformes [50], *Columba* [51], Buteoninae [52], Anatidae [53], and Apodidae [54]. Branch lengths (substitutions per site) were also obtained from these papers, but rescaled to correspond with the branch lengths in Hugall and Stuart-Fox [46]. Two versions of this phylogeny were used – the non-ultrametric composite tree using the raw branch length data (hereafter ‘Composite’), and the ultrametric tree (hereafter ‘Ultrametric’) produced using the semi-parametric penalized likelihood approach implemented in the *ape* package of R [55].

For the third phylogeny, we used a pruned version of the bird supertree from Davis [56]. As this tree does not contain branch length information, we opted to use equal branch lengths [57]. The final two phylogenies used were derived from the “Global Phylogeny of Birds” website – www.birdtree.org [58]. From this website we downloaded two sets of 2000 trees for our subset of species from the pseudo-posterior distribution of trees using the two available ‘backbones’ by Hackett et al. [47] and Ericson et al. [59]. We used these 2000 trees to calculate majority rule consensus phylogenies (hereafter the ‘Hackett’ and ‘Ericson’ phylogenies) using Mesquite [60]. Polytomies remaining in the phylogeny were arbitrarily resolved with internal branches assigned zero length. The five phylogenies are presented in File S1

### Phylogenetic comparative analysis

To correct for common ancestry, we used phylogenetic generalised least squares (PGLS) [61], as implemented in the R package *caper* [62]. First we calculated the amount of phylogenetic signal in individual traits using the maximum-likelihood value of the parameter λ [63], [64]. The phylogeny, with branch lengths, produces an expected variance-covariance matrix for the trait data which can then be compared to the observed covariance structure [44]. The calculated value is used as a multiplier of the off-diagonal elements in the variance-covariance matrix that best fits the observed data. In effect λ transforms the internal branch lengths of the phylogeny, When λ = 1, the internal branch lengths remain untransformed, indicating that the observed data strongly match expected phylogenetic patterns given a Brownian motion model of evolution. When λ = 0, all internal branches of the phylogeny collapse to zero, indicating there is no phylogenetic signal in the data.

For the PGLS regression calculations the maximum likelihood value of λ is calculated for the residual errors of the models (not the individual traits) and this value is used as the branch-length transformation in the subsequent GLS regression. Note that when λ = 0, the results are identical to analyses conducted using ordinary least squares regression on the raw data and when λ = 1 the results of PGLS will be identical to those obtained via Felsenstein's independent contrasts with an untransformed phylogeny [43]. In order to provide comparison with the recent analysis by Møller and Erritzøe [31], we also repeated the PGLS analysis with λ constrained to be 1 (i.e. equivalent to their independents contrasts analysis).

Brain size and eye size are closely correlated with body mass (r>0.8). To obtain a measure of the size of these organs and individual brain components that were independent of body size we calculated the residuals of the PGLS regression of the trait of interest against body mass (the observed value minus that predicted from the PGLS regression of the log-transformed trait on log body mass). For these calculations, rather than using species average body mass we used the body mass of the specific individuals in the original studies where brain size was measured [24], [31], [39].

### Model selection

We used a model selection approach to analyse the explanatory power of residual size of different brain regions, residual whole brain and eye sizes, and body mass and starting distance on FID. For each phylogeny, we compared models using Akaike's Information Criterion correcting for small sample size (AICc) [65], [66]. This approach allows comparisons of competing models with lower values of AIC representing ‘better’ models. The relative strength of each putative model is ascertained by calculating its Akaike weight (*w_i_*), which can be considered analogous to the probability that that model is the best approximating model. All multimodel inference and analysis was conducted using the *MuMIn* package in R [67].

We used the *dredge* function of *MuMIn* to compare models containing all combinations of the selected parameters. The exception to this was that we did not include relative whole brain size in the same analysis as individual brain regions, since the former is simply the sum of the latter. Instead we compared the Akaike score of the best model obtained using whole brain size as a predictor with the best model obtained using individual brain components as predictors to see which provided a better model for our data.

For each analysis we calculated the parameter weights (*w_(+j)_*) for each predictor (analogous to the probability that that predictor really does feature in best model), as well as weighted averages for the parameter estimates and 95% confidence intervals using the *model.avg* function in *MuMIn*. These estimates were then themselves averaged over the five different phylogenetic hypotheses to provide an overall estimate of the importance and nature of effect of each predictor on FID.

## Results

Individually, the traits used in the analyses generally exhibit strong phylogenetic signal (with the possible exception of relative brain stem size and relative optical lobe size) (Table 1). The phylogenetic generalised least squares analyses produced similar results irrespective on the phylogeny used as the basis for analysis (Table 2). In contrast to the phylogenetic signal estimates for the individual variables, the estimated maximum likelihood values of λ for the regression models were nearly always zero, indicating no phylogenetic signal in the residual errors of the models, and hence results that are equivalent to conventional ordinary least squares analyses. The small quantitative differences between phylogenies result from differences in residual values for brain (and brain components) derived from regressions of these variables against body mass where there was stronger phylogenetic signal (λ range  = 0.722–1.000 dependent on component and phylogeny).

**Table 1:** Phylogenetic signal estimates (maximum likelihood values of Pagel's λ) for individuals traits used in the analyses with values significantly different from zero (no phylogenetic signal) indicated in bold.

	Phylogeny used				
Trait	Composite	Ultrametric	Davis	Hackett	Ericson
Flight Initiation Distance	**0.886**	**0.855**	**0.962**	**0.847**	**0.845**
Starting Distance	**0.883**	**0.788**	**0.801**	**0.784**	**0.845**
Body mass	**1**	**1**	**1**	**1**	**1**
Brain size	**0.682**	**1**	**1**	**1**	**1**
Brain stem size	0.685	0.595	**0.837**	**0.658**	0.64
Optical lobe size	**0**	**0.692**	**0.615**	**0.802**	**0.822**
Cerebellum size	**0.935**	**0.909**	**1**	**0.969**	**0.981**
Forebrain size	**1**	**1**	**1**	**1**	**1**
Eye size	**1**	**1**	**1**	**1**	**1**

**Table 2:** Best models (ΔAICc <2) predicting Flight-Initiation Distance in birds as calculated from phylogenetic generalised least squares analyses using each of the five phylogenies.

Phylogeny		Model components	ΔAICc	w_i_	λ	R^2^ (%)
Composite	1	SD + Mass	0.00	0.18	0	68.83
	2	SD + Mass + Cereb	0.92	0.11	0	69.98
	3	SD + Mass + BStem + Cereb	1.11	0.10	0	71.69
		(SD + Mass + WholeBrain	2.40)			
Ultrametric	1	SD + Mass	0.00	0.15	0	65.53
	2	SD + Mass + Cereb	0.31	0.13	0	67.29
	3	SD + Mass + BStem + Cereb	0.72	0.11	0	69.00
		(SD + Mass + WholeBrain	2.02)			
Davis	1	SD + Mass	0.00	0.17	0	69.15
	2	SD + Mass + BStem + Cereb	0.85	0.11	0.6	61.05
	3	SD + Mass + Cereb	1.80	0.07	0	69.64
	4	SD + Mass + BStem	1.96	0.06	0	69.52
		(SD + Mass + WholeBrain)	2.46)			
Hackett	1	SD + Mass	0.00	0.19	0	65.81
	2	SD + Mass + Cereb	1.13	0.11	0	66.90
	3	SD + Mass + BStem + Cereb	1.52	0.09	0	68.64
		(SD + Mass + WholeBrain	2.32)			
Ericsson	1	SD + Mass	0.00	0.20	0	64.61
	2	SD + Mass + Cereb	1.36	0.10	0	65.55
	3	SD + Mass + BStem + Cereb	1.85	0.08	0	67.28
		(SD + Mass + WholeBrain	2.38)			

SD  =  Starting Distance, Mass  =  body mass, BStem  =  relative brain stem size, Cereb  =  relative cerebellum size, Foreb  =  relative forebrain size, Optic  =  relative optic lobe size, Eye  =  relative eye size, WholeBrain  =  relative whole brain size.

**Figure 1:**
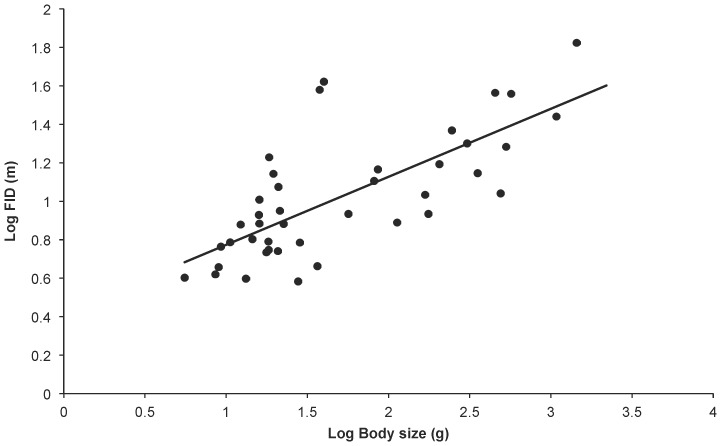
FID and body size. Relationship between log FID and log body mass for 41 bird species. The raw data are plotted with the phylogenetic generalised least squares regression line generated from the composite phylogeny with raw branch lengths.

**Figure 2:**
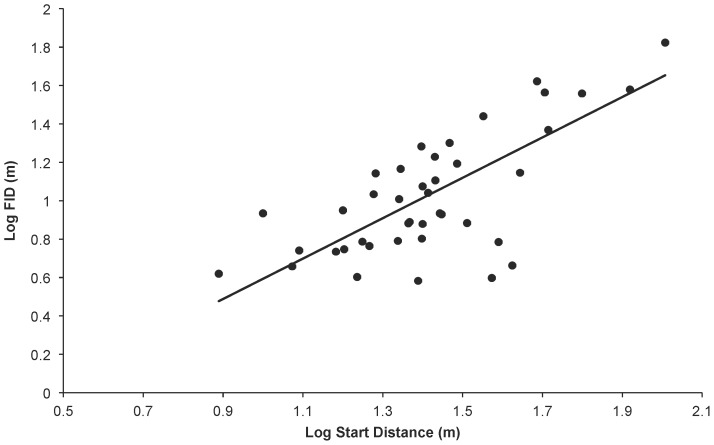
FID and Starting Distance. Relationship between log FID and log Starting Distance (the distance at which an approach to the bird was commenced) (both in m) for 41 bird species. The raw data are plotted with the phylogenetic generalised least squares regression line generated from the composite phylogeny with raw branch lengths.

There were broad aspects of agreement, however, in the PGLS analyses using all five phylogenies. First, starting distance and body size were the sole predictors found in all top models and were universally strongly weighted (cumulative weights for both were close to 100%, see Table 3). FID was strongly positively associated with both variables (Figures 1 and 2), and they explained approximately 65% of the variation in FID. Second, whole brain size was generally poorly weighted as a variable, and models featuring whole brain size received poor support. Models featuring individual brain components were more strongly weighted than the model featuring whole brain size.

**Table 3:** Averaged cumulative Akaike weights and coefficients for predictors of Flight-Initiation Distance calculated from the five phylogenies used in the analyses (see Table 1).

Predictor	w(_+j_)	Coefficient (95% CI)
Starting Distance	0.99	0.619 (0.300–0.937)
Body Mass	0.99	0.217 (0.104–0.329)
Brain Stem size	0.36	0.625 (−0.522–1.773)
Cerebellum size	0.44	−0.617 (−1.520–0.285)
Optic Lobe size	0.24	−0.121 (−0.885–0.642)
Forebrain size	0.25	−0.028 (−0.631–0.574)
Eye size	0.22	0.044 (−0.317–0.405)

**Figure 3:**
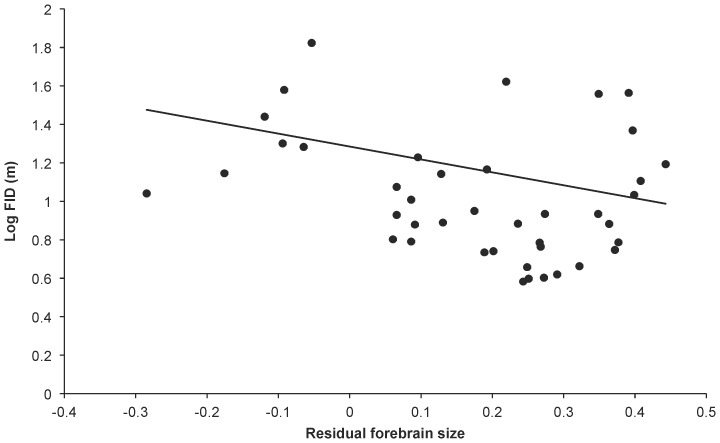
FID and forebrain size. Relationship between log FID and residual forebrain size for 41 bird species. The raw data are plotted with the phylogenetic generalised least squares regression line generated from the composite phylogeny with raw branch lengths.

Of all the individual brain components across all five PGLS analyses, cerebellum size has the strongest predictor weight (average  = 44%, Table 3). In the case of cerebellum size the relationship with FID is negative, indicating that birds with larger cerebellums are less ‘flighty’ (Figure 3), however the confidence intervals on the averaged cerebellum size estimate include zero. Other brain components feature less prominently in our credibility sets (Tables 3). It is notable that, in comparison to the large amount of variation explained by body size and starting distance, the addition of brain component variables, at best, only help explain an extra 3.5% of variation in FID.

The results from the analysis where λ is constrained to equal 1 (equivalent to independent contrasts with untransformed branch lengths), are substantially different from the PGLS analysis where λ adopts its maximum likelihood value (Tables 4 and 5). Here we found that individual brain components do feature in the top models, in particular relative forebrain size (negatively) in four top models (see Figure 3), and relative brain stem size (positively) in three top models. Starting distance was less strongly weighted in these analyses but features in three top models. Of all variables only body mass had an average estimate across all analyses whose confidence intervals excluded zero, although relative forebrain size had the next strongest predictor weight (average  = 71%) and the confidence intervals on its estimate only just included zero.

**Table 4:** Best models (ΔAICc <2) predicting Flight-Initiation Distance in birds as calculated from phylogenetic generalised least squares analyses where λ is constrained to equal 1 (equivalent to using independent contrasts analysis with the untransformed phylogenies).

Phylogeny		Model components	ΔAICc	w_i_	R^2^ (%)
Composite	1	Mass + Foreb	0.00	0.11	35.88
	2	SD + Mass + ForeB	1.05	0.07	38.05
	3	Mass + BStem +ForeB	1.18	0.06	37.86
	4	Mass + Optic + ForeB	1.47	0.05	37.42
	5	SD + Mass + BStem + ForeB	1.63	0.05	41.04
		(Mass + WholeBrain	5.00)		
Ultrametric	1	Mass + Foreb	0.00	0.12	32.02
	2	Mass + BStem + Foreb	0.46	0.10	35.27
	3	SD + Mass + BStem + Foreb	1.46	0.06	37.74
	4	SD + Mass + Foreb	1.63	0.05	33.39
		(Mass + WholeBrain	1.20)		
Davis	1	SD + Mass + BStem + Cereb	0.00	0.23	55.51
	2	SD + Mass + BStem + Cereb + ForeB	0.98	0.14	57.40
	3	SD + Mass + BStem + Cereb + Eye	1.98	0.09	56.34
		(SD + Mass + WholeBrain)	7.80)		
Hackett	1	SD + Mass + BStem + Foreb	0.00	0.09	38.29
	2	Mass + BStem + Foreb	0.22	0.08	33.90
	3	Mass + Foreb	0.23	0.08	29.78
	4	SD + Mass + Foreb	0.91	0.06	32.78
	5	SD + Mass + BStem + Cereb + Foreb	1.84	0.04	39.66
		(Mass + WholeBrain	1.20)		
Ericsson	1	SD + Mass + BStem + Forebrain	0.00	0.08	39.77
	2	Mass + Cereb + Forebrain	0.00	0.08	31.88
	3	Mass + Forebrain	0.04	0.08	31.79
	4	SD + Mass + Forebrain	0.87	0.05	34.45
	5	SD + Mass + BStem + Cereb + ForeB	1.83	0.03	41.11
		(Mass + WholeBrain	7.40)		

Abbreviations as per Table 2.

**Table 5:** Averaged cumulative Akaike weights and coefficients for predictors of Flight-Initiation Distance calculated from the five phylogenies using phylogenetic generalised least squares analyses where λ is constrained to equal 1 (equivalent to using independent contrasts analysis with the untransformed phylogenies).

Predictor	w(_+j_)	Coefficient (95% CI)
Starting Distance	0.57	0.353 (−.017–0.722)
Body Mass	0.92	0.292 (0.089–0.496)
Brain Stem size	0.53	0.993 (−0.308–2.295)
Cerebellum size	0.43	−0.717 (−1.919–0.485)
Optic Lobe size	0.25	0.126 (−0.757–1.008)
Forebrain size	0.71	−0.888 (−1.787–0.010)
Eye size	0.23	−0.135 (−0.613–0.342)

## Discussion

The greatest weight of evidence among the variables we tested was for starting distance and body mass to positively influence FID, both well-established relationships [1], [3], [4]. By comparison, the weight of evidence for, and effect size associated with, whole or part brain variables influencing FID was generally poor.

This study found little support for the contention that relative whole brain size influences FID. Similarly, Guay et al. [10] tested whether larger-brained shorebirds (25 species) had reduced FID in response to a frequently occurring benign stimulus (a walker) but found no effect of whole brain size. However, Carette and Tella [17] suggested that large brained bird species decrease their FIDs to cars in urban areas more readily because they exhibit greater between-individual variation in behaviour. In that case, relative brain size was correlated with bird's capacity to modify their fear response to humans (i.e., reduce FID). The ‘integrated brain’ argument suggests that many distributed parts of the brain are used in learning and decision making processes, and that relative whole brain size is a useful metric which is associated with a range of behavioural adaptations [30]. However, we found generally that models that include specific parts were better predictors than models with whole brain size used. This is in line with previous arguments that functional separation of brain components may be such that brain components are best considered separately in studies that attempt to link brain size and structure with behaviour [11]. However, overall, the phylogenetic generalised least squares analysis suggests no important effect of any brain component, or eye size, on FID.

The most notable aspect of these results is the extent to which they differ from those of a similar recent study by Møller and Erritzøe [31]. With the exception of finding a positive relationship of FID to body mass, our analysis suggests very different conclusions in regard to brain structures. We found no support for a link of FID with eye size or whole brain size. Neither did we find evidence of a positive relationship with cerebellum size. Although cerebellum size was the most strongly weighted brain component in our analysis, its importance was still weak, and the analysis suggests a negative relationship to FID. Given that our analyses mostly utilise a subset of the same data employed by Møller and Erritzøe (hereafter M&E), it raises the question of how we have derived such different results.

Two key differences lie in control variables we used in the analysis. In the case of eye size differences arise in the exact statistical measure of eye size used. Because eye size and body size were highly correlated (r = 0.87), we used residual eye size in model formulations, where M&E used the absolute log-transformed value – consequently it may not be surprising that we therefore fail to observe a positive relationship between eye size and FID. In absolute terms, it seems likely that eye size is linked with FID [24], [31].

A second, more fundamental, difference in the two analyses lies in the manner of controlling for starting distance. We recorded SD and controlled for its effects by including it as a continuous variable in analyses. This “statistical control” approach represents the commonest way of dealing with SD [4], [24], [32], but has been criticised as creating a mathematical artefact because FID can never exceed SD [68]. Although some analytical alternatives exist, they are not without constraints [69]. While Dumont et al. [68] recommended standardising SD, which is practically difficult, M&E and others [70], [71] “standardised” SD by ensuring approaches began from farther than a minimum distance (e.g., 30 m). Under such circumstances, the inclusion of SD does not alter the results of analyses and SD is excluded from models for simplicity [70]–[72]. M&E used a “stepped minimum” SD of *c.* 30 m for smaller species or *c.* 100 m for birds heavier than 150 g (apparently mostly estimated by eye), and report that the inclusion of SD in models did not substantively change them, thus SD was omitted from analyses. The mean (± standard deviation) SDs used by this study were 30.8±18.8 m (7.8–101.7 m). 68.3% were under 30 m, and so methodological differences may underpin some of the differences in findings. The relative merits and comparability of standardised and non-standardised approaches warrants further investigation, but could underpin some of the differences observed between this study and that of M&E.

Whilst both our analysis and M&E utilise phylogenetic comparative approaches, our analysis differed in testing for phylogenetic signal. Whilst individual traits showed strong phylogenetic signal, the error structure from the PGLS regression models did not, and indeed in every case the estimate for λ was strongly significantly different from 1 (the assumption of strong phylogenetic signal). It is worthwhile noting that this illustrates that it is not necessarily automatic that if there is strong phylogenetic signal in individual traits that this will remain for tests of associations between those traits [73]. By contrast M&E used independent contrasts with an approach that assumed a strong phylogenetic signal (i.e. no branch length transformation was applied). We observed considerable differences in our PGLS results when λ was constrained to equal 1 (i.e. replicating an independent contrasts analysis with untransformed branch lengths). In this case, results suggested that brain components do feature in the top models predicting FID, including possibly brain stem, forebrain and cerebellar sizes depending on the phylogeny used. However, in no case did these results reflect those of M&E. We again found a negative (not positive) relationship with cerebellum size, and a stronger negative effect with forebrain size (which was identified as having a non-significant effect by M&E).

Detailed mapping of the functions of parts of the avian brain is incomplete [23], however the cerebellum and forebrain are associated with, among other things, coordination of motor responses (including flight), and cognitive processing including perception of risk [21], [74]. Thus where relatively larger cerebella and forebrains are present, capacity for complex, and presumably effective, escape responses may be evident. Effective escape at shorter distances may permit birds to reduce the energetic and other costs of flight by delaying and sometimes avoiding flight. Most of the species in our analysis are those that have substantial exposure to humans. Since most human approaches are non-threatening then there could be a selective advantage to reduce FID in response to humans. The capacity to distinguish threat from non-threat must rely on learning, and it may be that a negative association between these brain components and FID reflects the greater capacity for species with large brain structures to process and learn that humans are non-threatening, and hence reduce FID. However, given the weak nature of the relationships, it would be unwise to draw too strong a conclusion as to the mechanism underlying any possible relationship. Additionally, despite the evidence for a stronger effect of forebrain indicated from the independent contrasts analysis, these models have considerably less support and less predictive capacity than models derived from PGLS analysis where the extent of phylogenetic signal was accounted for.

Whilst studies across taxa have shown significant relationships between brain components sizes and specific behaviours [75]–[77], the effect sizes are often only moderate (R-squared values of 10-20% in the case of the cited studies), and other studies which have looked for associations between ecology, behaviour and brain size in birds have found only equivocal support for associations [14], [22], [78]. Our results, in combination with an early study of whole brain size and FID in birds [10], indicate that any link between escape response behaviour and the size of brain structures is not strong, and may reflect the inherent difficulties and generalisations of relating complex behaviours with specific areas of the brain which themselves may be associated with a myriad of behavioural functions [11]. Data on brain composition from a greater number and diversity of species, ideally at a finer anatomical scale, would help elucidate if there truly is a biological significance of differences in flight behaviour in relation to the evolution of neural anatomy.

## Supporting Information

Table S1 **Bird FID and brain component data used in the analysis.**
(DOCX)

File S1 **Phylogenies used in the analysis for use in Nexus format.**
(DOCX)
