# Supplementary material for: Comparative Analysis of Classic Brain Component Sizes in Relation to Flightiness in Birds
Source: PLoS One. 2014 Mar 17;9(3):e91960. doi: 10.1371/journal.pone.0091960 (PMC3956822; doi:10.1371/journal.pone.0091960)
Supplement: Table S1 — Bird FID and brain component data used in the analysis. (DOCX) [file pone.0091960.s001.docx]

**Table S1.** Raw data used for the analyses. Presented are Start Distance (SD), Flight Initiation Distance (FID) (in m) along with sample size (n), body mass (BM, in g) and eye size (ES, volume in cm^3^) for each species. Brain component masses (in g) are shown for the Brain Stem (BS), Optical Lobe (OL), Cerebellum (C), Forebrain (F) and Whole Brain (WB).

| Family | Latin name | SD | FID | N | BM | ES | BS | OL | C | F | WB |
| --- | --- | --- | --- | --- | --- | --- | --- | --- | --- | --- | --- |
| Sylviidae | *Acrocephalus scirpaceus* | 25.1 | 7.6 | 4 | 12 | 0.074 | 0.065 | 0.070 | 0.066 | 0.282 | 0.483 |
| Aegithalidae | *Aegithalos caudatus* | 7.8 | 4.2 | 4 | 9 | 0.038 | 0.079 | 0.059 | 0.035 | 0.283 | 0.456 |
| Alaudidae | *Alauda arvensis* | 48.6 | 41.9 | 29 | 40 | 0.109 | 0.110 | 0.115 | 0.087 | 0.865 | 1.176 |
| Anatidae | *Anas platyrhynchos* | 35.6 | 27.6 | 29 | 1082 | 0.696 | 0.928 | 0.400 | 0.553 | 4.000 | 5.881 |
| Motacillidae | *Anthus pratensis* | 26.9 | 16.9 | 31 | 18 | 0.078 | 0.079 | 0.074 | 0.064 | 0.316 | 0.533 |
| Apodidae | *Apus apus* | 83.0 | 38.0 | 5 | 38 | 0.266 | 0.114 | 0.070 | 0.110 | 0.370 | 0.665 |
| Ardeidae | *Ardea cinerea* | 101.7 | 66.7 | 9 | 1443 | 2.142 | 1.155 | 0.749 | 0.996 | 4.967 | 7.867 |
| Fringillidae | *Carduelis cannabina* | 19.1 | 13.9 | 28 | 20 | 0.043 | 0.080 | 0.063 | 0.065 | 0.429 | 0.636 |
| Fringillidae | *Carduelis carduelis* | 32.5 | 7.7 | 4 | 16 | 0.036 | 0.079 | 0.049 | 0.070 | 0.421 | 0.619 |
| Fringillidae | *Carduelis spinus* | 37.4 | 4.0 | 5 | 13 | 0.037 | 0.069 | 0.054 | 0.069 | 0.354 | 0.546 |
| Certhiidae | *Certhia familiaris* | 11.8 | 4.6 | 3 | 9 | 0.029 | 0.074 | 0.047 | 0.050 | 0.277 | 0.447 |
| Fringillidae | *Coccothraustes coccothraustes* | 10.0 | 8.6 | 1 | 57 | 0.141 | 0.191 | 0.160 | 0.160 | 1.115 | 1.626 |
| Columbidae | *Columba livia* | 44.0 | 14.0 | 1 | 355 | 0.275 | 0.439 | 0.270 | 0.331 | 1.272 | 2.312 |
| Columbidae | *Columba palumbus* | 25.9 | 11.0 | 66 | 490 | 0.55 | 0.441 | 0.299 | 0.350 | 1.308 | 2.399 |
| Corvidae | *Corvus corone* | 63.0 | 36.2 | 35 | 570 | 0.88 | 0.748 | 0.531 | 0.675 | 6.429 | 8.383 |
| Corvidae | *Corvus frugilegus* | 50.8 | 36.7 | 44 | 454 | 0.87 | 0.670 | 0.475 | 0.640 | 6.005 | 7.790 |
| Corvidae | *Corvus monedula* | 51.8 | 23.4 | 32 | 246 | 0.548 | 0.410 | 0.360 | 0.396 | 3.593 | 4.758 |
| Hirundinidae | *Delichon urbicum* | 25.0 | 6.4 | 27 | 15 | 0.06 | 0.069 | 0.044 | 0.045 | 0.282 | 0.442 |
| Picidae | *Dendrocopos major* | 27.0 | 12.8 | 6 | 82 | 0.259 | 0.294 | 0.165 | 0.280 | 1.964 | 2.703 |
| Muscicapidae | *Erithacus rubecula* | 15.2 | 5.4 | 26 | 18 | 0.133 | 0.104 | 0.088 | 0.077 | 0.346 | 0.613 |
| Fringillidae | *Fringilla coelebs* | 15.8 | 8.9 | 67 | 21 | 0.086 | 0.094 | 0.091 | 0.079 | 0.466 | 0.731 |
| Rallidae | *Fulica atra* | 24.9 | 19.2 | 10 | 531 | 0.263 | 0.529 | 0.300 | 0.409 | 2.089 | 3.326 |
| Rallidae | *Gallinula chloropus* | 29.3 | 20.0 | 35 | 305 | 0.28 | 0.337 | 0.240 | 0.279 | 1.206 | 2.062 |
| Corvidae | *Garrulus glandarius* | 18.9 | 10.8 | 13 | 168 | 0.592 | 0.440 | 0.371 | 0.380 | 2.911 | 4.101 |
| Hirundinidae | *Hirundo rustica* | 21.9 | 10.2 | 25 | 16 | 0.077 | 0.072 | 0.055 | 0.082 | 0.342 | 0.551 |
| Laniidae | *Lanius collurio* | 38.9 | 6.1 | 2 | 28 | 0.266 | 0.115 | 0.106 | 0.112 | 0.719 | 1.052 |
| Fringillidae | *Loxia curvirostra* | 42.1 | 4.6 | 12 | 36 | 0.098 | 0.163 | 0.110 | 0.150 | 0.966 | 1.389 |
| Motacillidae | *Motacilla alba* | 25.1 | 11.9 | 30 | 21 | 0.08 | 0.085 | 0.085 | 0.082 | 0.374 | 0.626 |
| Muscicapidae | *Muscicapa striata* | 28.0 | 8.5 | 6 | 16 | 0.107 | 0.078 | 0.058 | 0.067 | 0.295 | 0.498 |
| Paridae | *Parus caeruleus* | 17.7 | 6.1 | 34 | 11 | 0.049 | 0.080 | 0.059 | 0.052 | 0.491 | 0.682 |
| Paridae | *Parus major* | 16.0 | 5.6 | 63 | 18 | 0.075 | 0.104 | 0.084 | 0.078 | 0.642 | 0.909 |
| Passeridae | *Passer domesticus* | 24.4 | 3.8 | 64 | 28 | 0.077 | 0.120 | 0.090 | 0.095 | 0.664 | 0.968 |
| Corvidae | *Pica pica* | 30.6 | 15.6 | 61 | 206 | 0.469 | 0.491 | 0.381 | 0.440 | 4.269 | 5.581 |
| Picidae | *Picus viridis* | 27.7 | 8.6 | 24 | 176 | 0.462 | 0.481 | 0.265 | 0.419 | 3.220 | 4.384 |
| Prunellidae | *Prunella modularis* | 12.3 | 5.5 | 22 | 21 | 0.088 | 0.108 | 0.097 | 0.081 | 0.440 | 0.725 |
| Reguliidae | *Regulus regulus* | 17.2 | 4.0 | 10 | 6 | 0.03 | 0.043 | 0.046 | 0.043 | 0.225 | 0.357 |
| Sittidae | *Sitta europaea* | 23.1 | 7.6 | 8 | 23 | 0.086 | 0.125 | 0.085 | 0.125 | 0.726 | 1.060 |
| Sturnidae | *Sturnus vulgaris* | 22.1 | 14.7 | 62 | 86 | 0.142 | 0.219 | 0.162 | 0.186 | 1.221 | 1.787 |
| Sylviidae | *Sylvia borin* | 21.8 | 6.2 | 34 | 18 | 0.096 | 0.089 | 0.074 | 0.070 | 0.353 | 0.586 |
| Troglodytidae | *Troglodytes troglodytes* | 18.5 | 5.8 | 30 | 9 | 0.043 | 0.086 | 0.058 | 0.055 | 0.305 | 0.504 |
| Turdidae | *Turdus merula* | 23.3 | 7.8 | 182 | 113 | 0.283 | 0.248 | 0.205 | 0.194 | 1.161 | 1.808 |
