# Supplementary material for: Comparative Analysis of Classic Brain Component Sizes in Relation to Flightiness in Birds
Source: PLoS One. 2014 Mar 17;9(3):e91960. doi: 10.1371/journal.pone.0091960 (PMC3956822; doi:10.1371/journal.pone.0091960)
Supplement: File S1 — Phylogenies used in the analysis for use in Nexus format. (DOCX) [file pone.0091960.s002.docx]

**File S1: Phylogenies used in the analysis in NEXUS format**

**1) Composite phylogeny**

#NEXUS

BEGIN TAXA;

TITLE Taxa;

DIMENSIONS NTAX=57;

TAXLABELS

Parus_caeruleus Parus_major Acrocephalus_scirpaceus Delichon_urbica Hirundo_rustica Aegithalos_caudatus Sylvia_borin Alauda_arvensis Sturnus_vulgaris Turdus_merula Erithacus_rubecula Muscicapa_striata Sitta_europaea Certhia_familiaris Troglodytes_troglodytes Regulus_regulus Prunella_modularis Passer_domesticus Motacilla_alba Anthus_pratensis Fringilla_coelebs Loxia_curvirostra Carduelis_carduelis Carduelis_spinus Carduelis_cannabina Coccothraustes_coccothraustes Lanius_collurio Garrulus_glandarius Pica_pica Corvus_monedula Corvus_corax Corvus_frugilegus Corvus_corone Oriolus_oriolus Falco_tinnunculus Alcedo_atthis Dendrocopos_major Picus_viridis Dryocopus_martius Upupa_epops Accipiter_nisus Buteo_buteo Vanellus_vanellus Gallinago_gallinago Numenius_arquata Larus_ridibundus Fulica_atra Gallinula_chloropus Porphyrio_porphyrio Ardea_cinerea Phalacrocorax_carbo Apus_apus Columba_livia Columba_palumbus Perdix_perdix Anas_crecca Anas_platyrhynchos

;

END;

BEGIN TREES;

Title 'Trees from "Patstree.nex"';

LINK Taxa = Taxa;

TRANSLATE

1 Parus_caeruleus,

2 Parus_major,

3 Acrocephalus_scirpaceus,

4 Delichon_urbica,

5 Hirundo_rustica,

6 Aegithalos_caudatus,

7 Sylvia_borin,

8 Alauda_arvensis,

9 Sturnus_vulgaris,

10 Turdus_merula,

11 Erithacus_rubecula,

12 Muscicapa_striata,

13 Sitta_europaea,

14 Certhia_familiaris,

15 Troglodytes_troglodytes,

16 Regulus_regulus,

17 Prunella_modularis,

18 Passer_domesticus,

19 Motacilla_alba,

20 Anthus_pratensis,

21 Fringilla_coelebs,

22 Loxia_curvirostra,

23 Carduelis_carduelis,

24 Carduelis_spinus,

25 Carduelis_cannabina,

26 Coccothraustes_coccothraustes,

27 Lanius_collurio,

28 Garrulus_glandarius,

29 Pica_pica,

30 Corvus_monedula,

31 Corvus_corax,

32 Corvus_frugilegus,

33 Corvus_corone,

34 Oriolus_oriolus,

35 Falco_tinnunculus,

36 Alcedo_atthis,

37 Dendrocopos_major,

38 Picus_viridis,

39 Dryocopus_martius,

40 Upupa_epops,

41 Accipiter_nisus,

42 Buteo_buteo,

43 Vanellus_vanellus,

44 Gallinago_gallinago,

45 Numenius_arquata,

46 Larus_ridibundus,

47 Fulica_atra,

48 Gallinula_chloropus,

49 Porphyrio_porphyrio,

50 Ardea_cinerea,

51 Phalacrocorax_carbo,

52 Apus_apus,

53 Columba_livia,

54 Columba_palumbus,

55 Perdix_perdix,

56 Anas_crecca,

57 Anas_platyrhynchos;

TREE * UNTITLED = [&R] ((((((((((1:0.04252,2:0.050168):0.071075,((3:0.122452,((4:0.053882,5:0.076252):0.070162,(6:0.118718,7:0.139365):0.005201):0.00281):0.008652,8:0.125454):0.009803):0.005773,((9:0.097515,(10:0.124558,(11:0.081527,12:0.071914):0.020769):0.006015):0.027596,(((13:0.121646,(14:0.128161,15:0.106684):0.005148):0.011793,16:0.15021):0.004586,(17:0.095897,(18:0.092725,((19:0.062225,20:0.06147):0.018558,(21:0.072745,((22:0.041242,((23:0.037246,24:0.027925):0.001823,25:0.028999):0.004354):0.034364,26:0.071223):0.011704):0.016861):0.003522):0.014441):0.028449):0.004145):0.003306):0.025052,((27:0.110012,((28:0.069591,29:0.046494):0.002349,(30:0.034386,(31:0.025668,(32:0.021992,33:0.018595):0.002925):0.007624):0.015502):0.031854):0.021094,34:0.122608):0.023942):0.15035,35:0.21855):0.0093,(((36:0.3596,(37:0.152017,(38:0.11236,39:0.122275):0.069399):0.158626):0.0093,40:0.5177):0.031,(41:0.095489,42:0.065427):0.060123):0.0031):0.01085,(43:0.131392,((44:0.13485,45:0.10546):0.063967,46:0.140036):0.013831):0.024204):0.0062,(((47:0.042278,48:0.078516):0.090595,49:0.211388):0.265745,(50:0.1488,51:0.1829):0.0155):0.0124):0.003875,(52:0.384865,(53:0.198917,54:0.1736):0.094033):0.0062):0.1023,(55:0.790682,(56:0.036813,57:0.033131):0.224556):0.1023);

END;

**2. Ultrametric phylogeny**

#NEXUS

BEGIN TAXA;

TITLE Taxa;

DIMENSIONS NTAX=57;

TAXLABELS

Parus_caeruleus Parus_major Acrocephalus_scirpaceus Delichon_urbica Hirundo_rustica Aegithalos_caudatus Sylvia_borin Alauda_arvensis Sturnus_vulgaris Turdus_merula Erithacus_rubecula Muscicapa_striata Sitta_europaea Certhia_familiaris Troglodytes_troglodytes Regulus_regulus Prunella_modularis Passer_domesticus Motacilla_alba Anthus_pratensis Fringilla_coelebs Loxia_curvirostra Carduelis_carduelis Carduelis_spinus Carduelis_cannabina Coccothraustes_coccothraustes Lanius_collurio Garrulus_glandarius Pica_pica Corvus_monedula Corvus_corax Corvus_frugilegus Corvus_corone Oriolus_oriolus Falco_tinnunculus Alcedo_atthis Dendrocopos_major Picus_viridis Dryocopus_martius Upupa_epops Accipiter_nisus Buteo_buteo Vanellus_vanellus Gallinago_gallinago Numenius_arquata Larus_ridibundus Fulica_atra Gallinula_chloropus Porphyrio_porphyrio Ardea_cinerea Phalacrocorax_carbo Apus_apus Columba_livia Columba_palumbus Perdix_perdix Anas_crecca Anas_platyrhynchos

;

END;

BEGIN TREES;

Title 'Trees from "chronoPatstree.nex"';

LINK Taxa = Taxa;

TRANSLATE

1 Parus_caeruleus,

2 Parus_major,

3 Acrocephalus_scirpaceus,

4 Delichon_urbica,

5 Hirundo_rustica,

6 Aegithalos_caudatus,

7 Sylvia_borin,

8 Alauda_arvensis,

9 Sturnus_vulgaris,

10 Turdus_merula,

11 Erithacus_rubecula,

12 Muscicapa_striata,

13 Sitta_europaea,

14 Certhia_familiaris,

15 Troglodytes_troglodytes,

16 Regulus_regulus,

17 Prunella_modularis,

18 Passer_domesticus,

19 Motacilla_alba,

20 Anthus_pratensis,

21 Fringilla_coelebs,

22 Loxia_curvirostra,

23 Carduelis_carduelis,

24 Carduelis_spinus,

25 Carduelis_cannabina,

26 Coccothraustes_coccothraustes,

27 Lanius_collurio,

28 Garrulus_glandarius,

29 Pica_pica,

30 Corvus_monedula,

31 Corvus_corax,

32 Corvus_frugilegus,

33 Corvus_corone,

34 Oriolus_oriolus,

35 Falco_tinnunculus,

36 Alcedo_atthis,

37 Dendrocopos_major,

38 Picus_viridis,

39 Dryocopus_martius,

40 Upupa_epops,

41 Accipiter_nisus,

42 Buteo_buteo,

43 Vanellus_vanellus,

44 Gallinago_gallinago,

45 Numenius_arquata,

46 Larus_ridibundus,

47 Fulica_atra,

48 Gallinula_chloropus,

49 Porphyrio_porphyrio,

50 Ardea_cinerea,

51 Phalacrocorax_carbo,

52 Apus_apus,

53 Columba_livia,

54 Columba_palumbus,

55 Perdix_perdix,

56 Anas_crecca,

57 Anas_platyrhynchos;

TREE * UNTITLED = [&R] ((((((((((1:0.1250047236,2:0.1250047236):0.1900322103,((3:0.2744157581,((4:0.1297633539,5:0.1297633539):0.1389805702,(6:0.2582820834,7:0.2582820834):0.01046184069):0.005671834008):0.01853018105,8:0.2929459392):0.02209099466):0.01389503808,((9:0.2560023416,(10:0.2427743723,(11:0.1917382501,12:0.1917382501):0.05103612226):0.01322796931):0.06534373882,(((13:0.2757743989,(14:0.264230849,15:0.264230849):0.01154354991):0.02637644888,16:0.3021508478):0.009848133393,(17:0.2445205693,(18:0.2115111086,((19:0.1568613301,20:0.1568613301):0.046556669,(21:0.1672716473,((22:0.0765975879,((23:0.06387162924,24:0.06387162924):0.003536195896,25:0.06740782514):0.009189762764):0.06731031064,26:0.1439078985):0.02336374871):0.03614635183):0.008093109526):0.03300946064):0.06747841195):0.009347099252):0.007585891444):0.05767821255,((27:0.2696033946,((28:0.1670791333,29:0.1670791333):0.006728883965,(30:0.1198090432,(31:0.09173228632,(32:0.08029132927,33:0.08029132927):0.01144095706):0.02807675688):0.05399897405):0.09579537731):0.05561243073,34:0.3252158253):0.06139435917):0.3517310455,35:0.73834123):0.02493958207,(((36:0.6831996464,(37:0.3551380728,(38:0.222457342,39:0.222457342):0.1326807308):0.3280615736):0.01806258597,40:0.7012622323):0.05434344895,(41:0.4809984505,42:0.4809984505):0.2746072308):0.00767513073):0.02673302042,(43:0.698023102,((44:0.4175695034,45:0.4175695034):0.2264104376,46:0.6439799409):0.05404316108):0.09199073042):0.01589533483,(((47:0.1292057021,48:0.1292057021):0.1873444687,49:0.3165501708):0.4614801857,(50:0.7242107788,51:0.7242107788):0.05381957762):0.02787881083):0.008075231862,(52:0.800174878,(53:0.543919165,54:0.543919165):0.256255713):0.01380952117):0.1860156009,(55:0.8408293533,(56:0.1164544253,57:0.1164544253):0.724374928):0.1591706467);

END;

**3. Davis phylogeny**

#NEXUS

BEGIN TAXA;

TITLE Taxa;

DIMENSIONS NTAX=57;

TAXLABELS

Accipiter_nisus Acrocephalus_scirpaceus Aegithalos_caudatus Alauda_arvensis Alcedo_atthis Anas_crecca Anas_platyrhynchos Anthus_pratensis Apus_apus Ardea_cinerea Buteo_buteo Carduelis_cannabina Carduelis_carduelis Carduelis_spinus Certhia_familiaris Coccothraustes_coccothraustes Columba_livia Columba_palumbus Corvus_corax Corvus_corone Corvus_frugilegus Corvus_monedula Delichon_urbica Dendrocopos_major Dryocopus_martius Erithacus_rubecula Falco_tinnunculus Fringilla_coelebs Fulica_atra Gallinago_gallinago Gallinula_chloropus Garrulus_glandarius Hirundo_rustica Lanius_collurio Larus_ridibundus Loxia_curvirostra Motacilla_alba Muscicapa_striata Numenius_arquata Oriolus_oriolus Parus_caeruleus Parus_major Passer_domesticus Perdix_perdix Phalacrocorax_carbo Pica_pica Picus_viridis Porphyrio_porphyrio Prunella_modularis Regulus_regulus Sitta_europaea Sturnus_vulgaris Sylvia_borin Troglodytes_troglodytes Turdus_merula Upupa_epops Vanellus_vanellus

;

END;

BEGIN TREES;

Title All_species;

LINK Taxa = Taxa;

TRANSLATE

1 Accipiter_nisus,

2 Acrocephalus_scirpaceus,

3 Aegithalos_caudatus,

4 Alauda_arvensis,

5 Alcedo_atthis,

6 Anas_crecca,

7 Anas_platyrhynchos,

8 Anthus_pratensis,

9 Apus_apus,

10 Ardea_cinerea,

11 Buteo_buteo,

12 Carduelis_cannabina,

13 Carduelis_carduelis,

14 Carduelis_spinus,

15 Certhia_familiaris,

16 Coccothraustes_coccothraustes,

17 Columba_livia,

18 Columba_palumbus,

19 Corvus_corax,

20 Corvus_corone,

21 Corvus_frugilegus,

22 Corvus_monedula,

23 Delichon_urbica,

24 Dendrocopos_major,

25 Dryocopus_martius,

26 Erithacus_rubecula,

27 Falco_tinnunculus,

28 Fringilla_coelebs,

29 Fulica_atra,

30 Gallinago_gallinago,

31 Gallinula_chloropus,

32 Garrulus_glandarius,

33 Hirundo_rustica,

34 Lanius_collurio,

35 Larus_ridibundus,

36 Loxia_curvirostra,

37 Motacilla_alba,

38 Muscicapa_striata,

39 Numenius_arquata,

40 Oriolus_oriolus,

41 Parus_caeruleus,

42 Parus_major,

43 Passer_domesticus,

44 Perdix_perdix,

45 Phalacrocorax_carbo,

46 Pica_pica,

47 Picus_viridis,

48 Porphyrio_porphyrio,

49 Prunella_modularis,

50 Regulus_regulus,

51 Sitta_europaea,

52 Sturnus_vulgaris,

53 Sylvia_borin,

54 Troglodytes_troglodytes,

55 Turdus_merula,

56 Upupa_epops,

57 Vanellus_vanellus;

TREE * UNTITLED = [&R] ((44:1.0,(7:1.0,6:1.0):1.0):1.0,((17:1.0,18:1.0):1.0,(((48:1.0,(29:1.0,31:1.0):1.0):1.0,((27:1.0,(1:1.0,11:1.0):1.0):1.0,((10:1.0,45:1.0):1.0,(57:1.0,((30:1.0,39:1.0):1.0,35:1.0):1.0):1.0):1.0):1.0):1.0,(9:1.0,(((5:1.0,56:1.0):1.0,(47:1.0,(25:1.0,24:1.0):1.0):1.0):1.0,((40:1.0,(34:1.0,(32:1.0,(46:1.0,(22:1.0,(20:1.0,(19:1.0,21:1.0):1.0):1.0):1.0):1.0):1.0):1.0):1.0,(((42:1.0,41:1.0):1.0,(4:1.0,(3:1.0,((2:1.0,53:1.0):1.0,(23:1.0,33:1.0):1.0):1.0):1.0):1.0):1.0,((((51:1.0,(15:1.0,54:1.0):1.0):1.0,(52:1.0,((38:1.0,26:1.0):1.0,55:1.0):1.0):1.0):1.0,50:1.0):1.0,(49:1.0,((8:1.0,37:1.0):1.0,(43:1.0,(28:1.0,(12:1.0,(16:1.0,(14:1.0,(36:1.0,13:1.0):1.0):1.0):1.0):1.0):1.0):1.0):1.0):1.0):1.0):1.0):1.0):1.0):1.0):1.0):1.0):1.0;

END;

**4. Hackett phylogeny**

#NEXUS

BEGIN TAXA;

TITLE Taxa;

DIMENSIONS NTAX=41;

TAXLABELS

Anas_platyrhynchos Apus_apus Columba_palumbus Columba_livia Ardea_cinerea Fulica_atra Gallinula_chloropus Picus_viridis Dendrocopos_major Lanius_collurio Garrulus_glandarius Pica_pica Corvus_monedula Corvus_corone Corvus_frugilegus Parus_caeruleus Parus_major Alauda_arvensis Acrocephalus_scirpaceus Sylvia_borin Aegithalos_caudatus Delichon_urbica Hirundo_rustica Regulus_regulus Sitta_europaea Troglodytes_troglodytes Certhia_familiaris Sturnus_vulgaris Turdus_merula Erithacus_rubecula Muscicapa_striata Prunella_modularis Passer_domesticus Motacilla_alba Anthus_pratensis Fringilla_coelebs Coccothraustes_coccothraustes Carduelis_carduelis Loxia_curvirostra Carduelis_spinus Carduelis_cannabina

;

END;

BEGIN TREES;

Title Imported_trees;

LINK Taxa = Taxa;

TRANSLATE

1 Anas_platyrhynchos,

2 Apus_apus,

3 Columba_palumbus,

4 Columba_livia,

5 Ardea_cinerea,

6 Fulica_atra,

7 Gallinula_chloropus,

8 Picus_viridis,

9 Dendrocopos_major,

10 Lanius_collurio,

11 Garrulus_glandarius,

12 Pica_pica,

13 Corvus_monedula,

14 Corvus_corone,

15 Corvus_frugilegus,

16 Parus_caeruleus,

17 Parus_major,

18 Alauda_arvensis,

19 Acrocephalus_scirpaceus,

20 Sylvia_borin,

21 Aegithalos_caudatus,

22 Delichon_urbica,

23 Hirundo_rustica,

24 Regulus_regulus,

25 Sitta_europaea,

26 Troglodytes_troglodytes,

27 Certhia_familiaris,

28 Sturnus_vulgaris,

29 Turdus_merula,

30 Erithacus_rubecula,

31 Muscicapa_striata,

32 Prunella_modularis,

33 Passer_domesticus,

34 Motacilla_alba,

35 Anthus_pratensis,

36 Fringilla_coelebs,

37 Coccothraustes_coccothraustes,

38 Carduelis_carduelis,

39 Loxia_curvirostra,

40 Carduelis_spinus,

41 Carduelis_cannabina;

TREE Resolved = (1:100.5975447994999,((2:82.87009823200003,((3:11.038886917499985,4:11.038886923999963):32.83479722650006,(5:72.90274400249992,(6:14.500632112499977,7:14.50063210399999):29.20105597850002):1.9024875247500013):6.092070219658975):0.0,((8:16.321252447999996,9:16.321252429500003):32.19504110174999,((10:26.258171094999977,(11:16.186576293499996,(12:15.250126029999985,(13:10.11774667050001,(14:6.955601490000002,15:6.95560148):3.1621452005000004):2.588567012749999):0.5170478163209605):10.005277472499987):12.136789055499996,(((16:19.038433680000022,17:19.038433701500015):11.641909027499999,(18:37.264962921499944,((19:28.51475026449997,20:28.684284356999992):7.800027677419364,(21:29.80699343450002,(22:16.086809649999978,23:16.08680962149997):14.195688329999985):3.282084215024123):1.9017804165000003):5.057288772000005):1.1768535297500005,((24:43.227557559500006,((25:32.652209284499996,(26:29.03953980100002,27:29.03953982000002):1.8063347317499983):6.122269590999995,(28:29.454924641999966,(29:25.957328887999964,(30:18.01685751799998,31:18.016857518999963):7.940471366499993):3.4975957870000043):4.659777081500008):4.453078741500002):0.2589040517500003,(32:35.56823266300006,(33:31.777150628000033,((34:22.448462076000038,35:22.448462117500007):4.064876698750004,(36:26.037288955000008,(37:20.481807944999986,((38:8.412726663500004,39:8.516129132500009):0.42686799493243227,(40:7.155188986500005,41:7.155188980000003):1.8798655549999992):11.324477465999989):2.777740512249999):4.357577085999993):0.8027202411594193):1.8014585155):8.177132932999998):0.9305931079999997):5.855790396000003):30.179585572000008):18.53110354475003):17.31456196949999):0.0;

END;

**5. Ericson phylogeny**

#NEXUS

BEGIN TAXA;

TITLE Taxa;

DIMENSIONS NTAX=41;

TAXLABELS

Anas_platyrhynchos Columba_palumbus Columba_livia Ardea_cinerea Gallinula_chloropus Fulica_atra Apus_apus Picus_viridis Dendrocopos_major Lanius_collurio Garrulus_glandarius Pica_pica Corvus_monedula Corvus_frugilegus Corvus_corone Parus_major Parus_caeruleus Alauda_arvensis Acrocephalus_scirpaceus Sylvia_borin Aegithalos_caudatus Delichon_urbica Hirundo_rustica Regulus_regulus Sitta_europaea Troglodytes_troglodytes Certhia_familiaris Sturnus_vulgaris Turdus_merula Erithacus_rubecula Muscicapa_striata Prunella_modularis Passer_domesticus Motacilla_alba Anthus_pratensis Fringilla_coelebs Coccothraustes_coccothraustes Loxia_curvirostra Carduelis_carduelis Carduelis_spinus Carduelis_cannabina

;

END;

BEGIN TREES;

Title Imported_trees;

LINK Taxa = Taxa;

TRANSLATE

1 Anas_platyrhynchos,

2 Columba_palumbus,

3 Columba_livia,

4 Ardea_cinerea,

5 Gallinula_chloropus,

6 Fulica_atra,

7 Apus_apus,

8 Picus_viridis,

9 Dendrocopos_major,

10 Lanius_collurio,

11 Garrulus_glandarius,

12 Pica_pica,

13 Corvus_monedula,

14 Corvus_frugilegus,

15 Corvus_corone,

16 Parus_major,

17 Parus_caeruleus,

18 Alauda_arvensis,

19 Acrocephalus_scirpaceus,

20 Sylvia_borin,

21 Aegithalos_caudatus,

22 Delichon_urbica,

23 Hirundo_rustica,

24 Regulus_regulus,

25 Sitta_europaea,

26 Troglodytes_troglodytes,

27 Certhia_familiaris,

28 Sturnus_vulgaris,

29 Turdus_merula,

30 Erithacus_rubecula,

31 Muscicapa_striata,

32 Prunella_modularis,

33 Passer_domesticus,

34 Motacilla_alba,

35 Anthus_pratensis,

36 Fringilla_coelebs,

37 Coccothraustes_coccothraustes,

38 Loxia_curvirostra,

39 Carduelis_carduelis,

40 Carduelis_spinus,

41 Carduelis_cannabina;

TREE Resolved = (1:103.64047756799971,((2:11.2337282635,3:11.233728249000006):38.240124699750005,((4:74.72958711849998,(5:14.540773072000041,6:14.540773083000047):63.73926490799995):3.426368705999993,((7:82.93579390600004,(8:16.589413207499998,9:16.589413276499982):66.34638087350005):0.41184077550000014,((10:26.80953725000001,(11:16.545607370000035,(12:15.614989321500019,(13:10.397567077499984,(14:7.151693938499995,15:7.151693948499984):1.6229365732499983):2.631591267999992):1.0291366919715996):10.195056404499994):12.457284828499985,(((16:19.467442975999965,17:19.46744300049995):11.918373633250013,(18:38.248951262499965,((19:29.67530542500003,20:29.54480625400001):3.9154363860946755,(21:30.788821326499992,(22:16.723828450499962,23:16.723828465999972):7.3373199175):3.33052363169479):1.9145752879999993):5.055239044500008):1.1917733235000003,((24:44.19794021500005,((25:33.57566675999996,(26:29.730836533999994,27:29.73083656500002):1.9224150992499989):6.155832664000004,(28:30.11087422349998,(29:26.47040713849992,(30:18.344142746499987,31:18.34414277199999):8.126264403000008):3.6404671234999966):4.810312560000003):2.2332204322499964):0.26165690774999967,(32:36.26407002599999,(33:32.24675554999997,((34:22.718547749500047,35:22.718547741000034):4.199821115999992,(36:26.420789832500038,(37:20.79305787600003,((38:8.664074238999996,39:8.558578016999979):0.42819142709720376,(40:7.297049101499993,41:7.297049095000004):1.8966610950000002):11.477828808000002):2.8138659557500043):4.474577076000001):0.8079060279928532):1.8921601545000004):8.457183997000001):0.9664828544999997):6.036369943499999):15.811763930750038):1.7851407015):2.581202307500004):15.926499762999969):0.0;

END;
